# 

**Published:** 1842-05-28

**Authors:** 


### CONTENTS OF No. 22.

Dr. Reynell Coates' Contributions to Surgery:
Note on the Means of avoiding Excoriations of the Heel and Perineum in Fractures of the Lower Extremities, treated by Permanent Extension, - - - - - - - - 337
Dr. J. B. Brinton's case of Puerperal Convulsions, - - - - 340
Dr. M. W. Wilson's Clinical Report:
Large Vegetations of Mitral Valve—Pneumonia, &c. - - 341
ANALECTA: Cases illustrating the action of Benzoic Acid in Affections of the Urinary Organs, - - - - - - - - 343
Dr. Roberts' Case of Fatal Hæmorrhage from the extraction of a tooth; with the treatment, - - - - - 344
Dr. Levy on Inflammation of the Umbilical Arteries, as a cause of Trismus Neonatorum, - - - - - - - 347
Professor Renzi's Account af an Epidemic of Typhus, accompanied with Appoplectic and Tetanic Symptoms, &c. &c. 349
Dr. Fardel's Memoir on Softening of the Brain, - - - 351
M. Velpeau on the Hymen, - - - - - - - 352

